# Human METTL12 is a mitochondrial methyltransferase that modifies citrate synthase

**DOI:** 10.1002/1873-3468.12649

**Published:** 2017-04-27

**Authors:** Virginie F. Rhein, Joe Carroll, Shujing Ding, Ian M. Fearnley, John E. Walker

**Affiliations:** ^1^ Medical Research Council Mitochondrial Biology Unit University of Cambridge UK

**Keywords:** citrate synthase, metabolons, METTL12, mitochondria, protein methylation, substrate channelling

## Abstract

The protein methylome in mammalian mitochondria has been little studied until recently. Here, we describe that lysine‐368 of human citrate synthase is methylated and that the modifying enzyme, localized in the mitochondrial matrix, is methyltransferase‐like protein 12 (METTL12), a member of the family of 7β‐strand methyltransferases. Lysine‐368 is near the active site of citrate synthase, but removal of methylation has no effect on its activity. In mitochondria, it is possible that some or all of the enzymes of the citric acid cycle, including citrate synthase, are organized in metabolons to facilitate the channelling of substrates between participating enzymes. Thus, possible roles for the methylation of Lys‐368 are in controlling substrate channelling itself, or in influencing protein–protein interactions in the metabolon.

## Abbreviations

**ASMTL**, acetylserotonin O‐methyltransferase‐like

**COMTD1**, catechol O‐methyltransferase domain‐containing protein 1

**Ctm1p**, cytochrome c methyltransferase

**ECE2**, Endothelin‐converting enzyme 2

**EFM4**, elongation factor methyltransferase 4

**ETD**, electron transfer dissociation

**ETFβ**, β‐subunit of the electron transfer flavoprotein

**HAP1**, human haploid cell line 1

**HEK**, human embryonic kidney

**HEMK1**, heme biosynthesis gene K methyltransferase family member 1

**KMT2A**, histone‐lysine N‐methyltransferase 2A

**METTL10**, methyltransferase‐like protein 10

**METTL12**, methyltransferase‐like protein 12

**METTL13**, methyltransferase‐like protein 13

**METTL9**, methyltransferase‐like protein 9

**MTRF1L**, mitochondrial translation release factor 1‐like

**MTS**, mitochondrial targeting sequence

**NDUFAF7**, NADH dehydrogenase (ubiquinone) complex I assembly factor 7

**NDUFB3**, NADH dehydrogenase (ubiquinone) 1β subcomplex subunit 3

**NDUFS2**, NADH dehydrogenase (ubiquinone) iron–sulphur protein 2

**PRDM15**, positive regulatory domain‐containing protein 15

**PRMT8**, protein arginine methyltransferase 8

**RRNAD1**, ribosomal RNA adenine demethylase domain‐containing protein 1

**RSAD1**, radical *S*‐adenosyl methionine domain‐containing protein 1

**SAM**, *S*‐adenosyl‐methionine

**SET**, SET domain (*Drosophila* Su(var)3‐9, Enhancer of zeste and Trithorax)

**SETD4**, SET domain‐containing protein 4

**TOM and TIM**, translocases of the outer and inner mitochondrial membranes respectively

The methylation of lysine and arginine residues in nonhistone proteins is being associated increasingly with the regulation of many different cellular activities 1, 2. Lysine residues can also be ubiquitinated and sumoylated, and the interplay between methylation, acetylation and these other post‐translational modifications, at the same and neighbouring sites, adds complexity to the regulation of biological processes by post‐translational modification 3, 4, 5. Trimethylation of a lysine residue in mitochondrial apocytochrome c by Ctm1p in the cytoplasm of *Saccharomyces cerevisiae* is well known 6, 7, although the mammalian orthologue is unmodified. Methylation of yeast cytochrome c promotes its import into the mitochondrial intermembrane space of yeast mitochondria, and modulates its interaction with other proteins 8, 9. Four other major mitochondrial proteins have been reported to contain trimethylated lysines. They are citrate synthase 10 and the β‐subunit of the electron transfer flavoprotein (ETFβ) 11, both found in the mitochondrial matrix, the ADP/ATP translocase 12 and the c‐subunit in the rotor of the ATP synthase 13, both major components of the inner mitochondrial membrane. Two additional proteins are methylated in complex I. An arginine residue in the NDUFS2 subunit is symmetrically dimethylated 14, and three histidine residues near to the N terminus of the NDUFB3 subunit are methylated to varying extents 15. In addition to these sites characterized in proteins purified from mitochondria, other human proteins that locate to mitochondria have been reported in cell‐wide methylome studies to contain methylated lysine and arginine residues 16, 17, 18.

The methylation of proteins and RNA molecules is catalysed by methyltransferases with *S*‐adenosylmethionine as the methyl donor 19, 20. Until recently, the only human methyltransferases known to be located in mitochondria were HEMK1, which methylates a glutamine residue in the mitochondrial translation release factor MTRF1L 21, and NDUFAF7, an assembly factor for complex I 22. In a continuing systematic study of protein methylases in human mitochondria, we have shown that NDUFAF7 symmetrically dimethylates Arg‐85 in the NDUFS2 subunit of complex I, and that this modification is an essential step in the assembly of the complex 23. We have also demonstrated that METTL20 methylates lysine residues adjacent to the recognition loop of ETFβ 11. HEMK1, NDUFAF7 and METTL20 are all members of the cell‐wide protein family of 7β‐strand methyltransferases. This family is characterized by a common core similar to a Rossman fold with a twisted seven‐stranded β‐sheet structure and usually six associated α‐helices, although the number of α‐helices varies 19, 20. Another member of the family, NDUFAF5, also located in the matrix of mitochondria, hydroxylates Arg‐73 in subunit NDUFS7 of complex I 24. This modification is an essential early step in the assembly of the complex. Previously, we have confirmed that another 7β‐strand methyltransferase, METTL12, is found in the matrix of the organelle 11. Here, we demonstrate that METTL12 modifies Lys‐368 of citrate synthase in an external surface region close to its catalytic site.

## Materials and methods

### Bioinformatic analyses

The presence of N‐terminal mitochondrial targeting sequences (MTSs) in the 208 known and putative methyltransferases 25 was examined with MitoProt, iPSORT, TargetP and MitoFates 26, 27, 28, 29. The secondary structures of proteins were predicted with Jpred 30 and PSIPRED 31.

### Cell culture

Parental human embryonic kidney cells (HEK293T) and the same cells overexpressing METTL12 were grown under an atmosphere of 5% CO_2_ at 37 °C in high glucose (25 mm) Dulbecco's modified Eagle medium (DMEM) supplemented with tetracycline‐free FBS (10% v/v), penicillin (100 units·mL^−1^) and streptomycin (0.1 mg·mL^−1^). The medium also included blasticidin (15 μg·mL^−1^) and Zeocin (100 μg·mL^−1^), or blasticidin (15 μg·mL^−1^) and hygromycin (100 μg·mL^−1^) respectively. A human haploid cell line, HAP1 (Horizon Discovery), derived from a chronic myelogenous leukaemia, was grown at 37 °C in the presence of 5% CO_2_ in Iscove's modified Dulbecco's medium (IMDM) containing 10% (v/v) FBS, penicillin (100 units·mL^−1^) and streptomycin (0.1 mg·mL^−1^). A cell line, HAP1‐ΔMETTL12 (Horizon Discovery HZGHC000536c011), derived from the parental cells by disruption of *METTL12* by CRISPR‐Cas9 technology was grown under the same conditions. The proliferation of cells was assayed at 37 °C in a humidified atmosphere containing 5% CO_2_ with an IncuCyte HD instrument (Essen Bioscience, Welwyn Garden City, UK). Initially, 1 × 10^5^ parental HAP1 cells or HAP1‐ΔMETTL12 cells were seeded into three wells in a six‐well plate. Cell proliferation was followed by measuring confluence over a period of time and quantified with incucyte software (version 2011A; Essen Bioscience).

### Expression of tagged METTL12

The cDNA for human METTL12 (Source BioScience, Cambridge, UK) was amplified by PCR with the forward and reverse primers 5′‐ACCGAGCTCGGATCCGGAATGGCCGCGCTGCGTCGAAT‐3′ and 5′‐ACCGGTTCCCTCGAGATGAGAGCCTTGAATCAAGTAAG‐3′, respectively, which introduced Flag and Strep II tags at its C terminus. The amplified sequence was cloned into plasmid pcDNA5/FRT/TO. This plasmid and plasmid pOG44 were cotransfected in the presence of lipofectamine 2000 (Life Technologies, Paisley, UK) into human HEK293T Flp‐In T‐Rex cells (total DNA 1 μg; pOG44:pcDNA5/FRT/TO, 7 : 1 by weight) (Life Technologies) 32. After 24 h, the cells were transferred into selective medium containing blasticidin (15 μg·mL^−1^) and hygromycin (100 μg·mL^−1^), and inducible cell lines expressing the recombinant protein were selected. Expression of tagged METTL12 was induced for 24 h with doxycycline (20 ng·mL^−1^), then mitochondria 32 or mitoplasts 11, 33 were prepared from the cells.

### Protein analyses

Samples of proteins were fractionated by SDS/PAGE on Novex 10–20% acrylamide gradient gels (Life Technologies) and then transferred by electrophoresis to Immobilon P membranes (Millipore, Watford, UK). Membranes were washed with a solution (PBST) made from PBS containing Tween‐20 (0.01%, v/v), and then blocked with a solution of dried skimmed milk in PBST, or, when a methyl‐specific antibody was used, in PBST plus bovine serum albumin. Then, the membrane was exposed to the primary antibody. The primary antibodies were specific for methyllysine and trimethyllysine (Abcam, Cambridge, UK; ab23366 and ab76118), human mitochondrial malate dehydrogenase, citrate synthase and ETFβ (Proteintech, Manchester, UK; 15462‐1‐AP, 16131‐1‐AP and 17925‐1‐AP respectively). Bound primary antibodies were detected by chemiluminescence generated with an anti‐rabbit secondary antibody conjugated to horse radish peroxidase (Thermo Fisher Scientific, Loughborough, UK) and ECL prime reagents (GE Healthcare, Little Chalfont, UK). Bound proteins were stained with Coomassie Blue R250 0.08% (w/v) dye dissolved in an aqueous solution containing 20% methanol (v/v) and 2.8% acetic acid (v/v). The membrane was washed with 50% methanol (v/v) and dried in air. Then it was wetted with 100% methanol and reprobed with another antibody.

### Mass spectrometry

Samples were fractionated by SDS/PAGE as above, and proteins were detected by staining with Coomassie blue dye. The stained bands were excised, digested ‘in‐gel’ 34 with trypsin or AspN protease, and the peptides were analysed in an LTQ OrbiTrap XL‐electron transfer dissociation (ETD) mass spectrometer (Thermo Scientific, Hemel Hempstead, UK) coupled with a Proxeon Easy‐nLC (Thermo Scientific) for nanoscale reverse‐phase peptide separation, as described before 11, 23. Proteins were identified by interrogation of a human protein sequence database with the peptide mass and fragmentation data, with the aid of Proteome Discoverer 1.3 (Thermo Scientific) and the Mascot search engine, version 2.4.0 35. The identities of peptides containing methylated lysine residues, and the specific site of methylation, were determined by manual interpretation of ETD fragmentation data. Fragment ions generated by ETD are labelled as c and z‐ions, with the related z+H‐ions included with z‐ions 36. The relative abundances of peptides bearing one, two, three or no methyl groups were determined with Xcalibur data analysis software from calculated peak areas of Gaussian smoothed extracted ion chromatograms, applying a 5 p.p.m. *m*/*z* tolerance and overlapping peptide retention times.

### Assay of citrate synthase

The activity of citrate synthase in parental HAP1 cells and HAP1‐ΔMETTL12 cells was determined by an immunocapture‐based microplate assay (Abcam) requiring the measurement of thionitrobenzoic acid released by the reaction of 5,5‐dithiobis‐(2‐nitrobenzoic acid) with CoA‐SH produced by the enzymic generation of citrate from oxaloacetate and acetyl‐CoA 37. The concentration of citrate synthase in cell lysates was determined with an ELISA assay kit (Elabscience, Wuhan, China). Both assays were performed in a Spectramax Plus 384 microplate reader (Molecular Devices, Wokingham, UK), and analysed with softmax pro software (Molecular Devices).

### Immunocapture of citrate synthase

Mitochondria from wild‐type HAP1 cells and HAP1‐ΔMETTL12 cells, were solubilized at a protein concentration of 5 mg·mL^−1^ with 1% (w/v) dodecylmaltoside prepared in PBS containing complete protease inhibitors (Roche, Mannheim, Germany). An anti‐citrate synthase monoclonal antibody (Abcam ab128564) was added (5 μL per 100 μL of mitochondrial solution), and the sample was kept at 4 °C for 4 h. A 50% (w/v) slurry of protein‐G agarose (10 μL; Abcam) was added, and the sample was mixed for 4 h at 4 °C. Unbound material was removed, and the agarose was washed four times with PBS containing 0.1% dodecylmaltoside. Bound proteins were eluted with a solution of 0.2 m glycine‐HCl, pH 2.5, and neutralized with a 1 m Tris solution to a final concentration of ca. 130 mm.

## Results

### Mitochondrial protein methyltransferases

Previously, a catalogue of known and predicted methyltransferases in human mitochondria has been presented 23. The entries were selected from a larger catalogue of 208 known and putative methyltransferases 25 on the basis of their having an N‐terminal sequence with the characteristic features of a mitochondrial targeting signal that would be removed by proteolysis during import of the protein into the organelle, to generate the mature protein in the matrix of the mitochondrion 38. This past catalogue contained not only known protein methyltransferases but also methyltransferases that modify RNA, and others involved in the biogenesis of small molecules. The new catalogue presented here (Table 1) is restricted to known protein methyltransferases and unknown predicted methyltransferases. Thus, potentially, some unknown entries could modify proteins, and others either RNA or small molecules. Four proteins, PRMT8, PRMT9, PRDM15 and KMT2A, are all predicted by two programs to be mitochondrial proteins, but PRMT8 is a plasma membrane protein 39 and the others are found in the nucleus and/or have been shown to methylate nuclear proteins 40, 41, 42, 43. Therefore, they have been excluded from the new catalogue. Another potential methyltransferase, SETD4, has been described as being a component of both the nucleus and the cytoplasm 44. As the cytoplasmic attribution could conceivably represent the mitochondria, SETD4 remains in this new restricted catalogue.

**Table 1:** Known and possible protein methyltransferases in human mitochondria

Protein	Family^a^	MTS^b^	Function^c^	Target^d^	Location^e^	References
HEMK1	7β	1	Gln‐Me	PRF1‐L	M	21, 63
METTL12	7β	3	Lys‐Me	CS	M	This work
METTL20	7β	3	Lys‐Me	ETFβ	M	11, 45
NDUFAF5	7β	4	Arg‐OH	NDUFS7	M	24, 61, 63
NDUFAF7	7β	4	Arg‐Me	NDUFS2	M	23, 62, 63
SETD9	SET	4	Unknown	Unknown	M	64
RSAD1	R‐SAM	4	Unknown	Unknown	M	64
COMTD1	7β	1	Unknown	Unknown	M	63
SETD4	SET	2	Unknown	Unknown	N, C	44
METTL9	7β	4	Unknown	Unknown	Unknown	
ASMTL	7β	3	Unknown	Unknown	Unknown	
RRNAD1	7β	4	Unknown	Unknown	Unknown	

^a^7β, 7β‐strand; SET, SET domain (*Drosophila* Su(var)3‐9, Enhancer of zeste and Trithorax); R‐SAM, radical *S*‐adenosylmethionine. ^b^MTS, number of independent predictions of the presence of a N‐terminal mitochondrial targeting sequence; unless there is experimental evidence for their localization in mitochondria, singly predicted proteins have been excluded. ^c^Gln‐Me, Lys‐Me and Arg‐Me, glutamine, lysine and arginine methyltransferases respectively; Arg‐OH, arginine hydroxylase. ^d^PRF1‐L, peptide release factor 1‐like; ETFβ, the β‐subunit of the electron transfer flavoprotein; CS, citrate synthase. ^e^Subcellular location of methyltransferase; M, mitochondria; N, nucleus; C, cytoplasm.

### Overexpression of METTL12 and methylation of human citrate synthase

**Figure 1:**
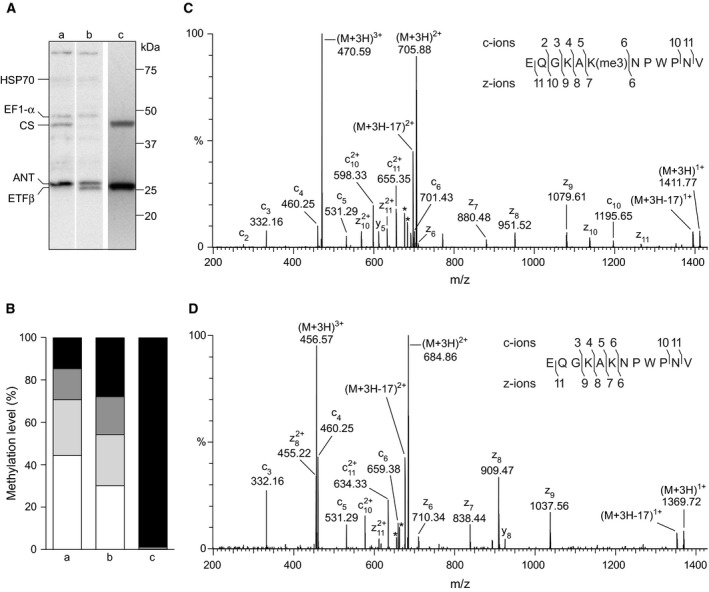
The methylation of human citrate synthase accompanying overexpression of METTL12. (A) Fractionation by SDS/PAGE of mitochondria from HEK293T cells overexpressing, in track a, METTL12, and in tracks b and c, METTL20. Proteins in tracks a and b were detected with an antibody recognizing methylated lysine residues, and track c is track b, stripped of bound antibodies and reprobed with antibodies against citrate synthase (CS) and ETFβ. The positions of the proteins containing trimethylated lysine residues are indicated on the left; HSP70, 70‐kDa heat shock protein; EF1α, elongation factor 1α; ANT, ADP/ATP translocase. (B) The relative peak areas taken from the extracted ion chromatograms of the AspN peptide EQGKAKNPWPNV (residues 363–374) of citrate synthase in mitoplast samples, containing unmethylated, and mono‐, di‐ and trimethylated lysine‐368 residues (white, light grey, dark grey and black areas respectively). Column a, control cells; b and c, cells overexpressing METTL20 and METTL12 respectively. (C,D) Fragmentation spectra of the AspN peptide produced by ETD of triply charged ions with *m*/*z* 470.59 and 456.57 respectively. The asterisks indicate ions arising from neutral losses of CH_3_ NO and C_2_H_5_ NO from Gln and Asn residues. In the insets, fragment ions have been mapped onto the amino acid sequence.

METTL12, was overexpressed in HEK293T cells, and, in comparison to an equivalent cell line overexpressing a second mitochondrial methyltransferase, METTL20, that modifies two lysine residues in ETFβ 11, 45, the overexpression of METTL12 was accompanied by an increase in the methylation of a lysine residue (or residues) in citrate synthase (Fig. 1). In order to identify the methylated site or sites, peptides in AspN and trypsin digests of human citrate synthase were analysed by mass spectrometry from the samples where METTL12 and METTL20 had been overexpressed (Tables S1–S4). The numbering of residues in the sequence of citrate synthase refers to the mature protein produced by removal of the N‐terminal 27 amino acid MTS 10. The fragmentation spectra of two triply charged ions of an AspN peptide corresponding to residues 363–374, with *m*/*z* values of 470.59 and 456.57, correspond to trimethylated and unmethylated states respectively. These fragmentation spectra contained the ions c_5_‐c_6_ and z_6_‐z_7_, which show that residue Lys‐368 is trimethylated (Fig. 1), whereas the ions c_3_‐c_4_ and z_8_‐z_9_ show Lys‐366 is unmodified. From the relative abundances of the mono‐, di‐ and trimethylated forms of the peptide (Table S5), it was calculated that 99% of this residue was trimethylated in cells overexpressing METTL12, in contrast to 14.7% of the residue being trimethylated in control cells. In the same control cells, the abundances of dimethylated, monomethylated and unmethylated states were 14.6%, 26.4% and 44.3% respectively (Fig. 1). Similar levels of partial methylation of this site were observed in cells overexpressing METTL20 (Fig. 1), which has no effect on citrate synthase, but instead modifies two lysine residues in ETFβ 11, 45.

### Impact of disruption of METTL12

**Figure 2:**
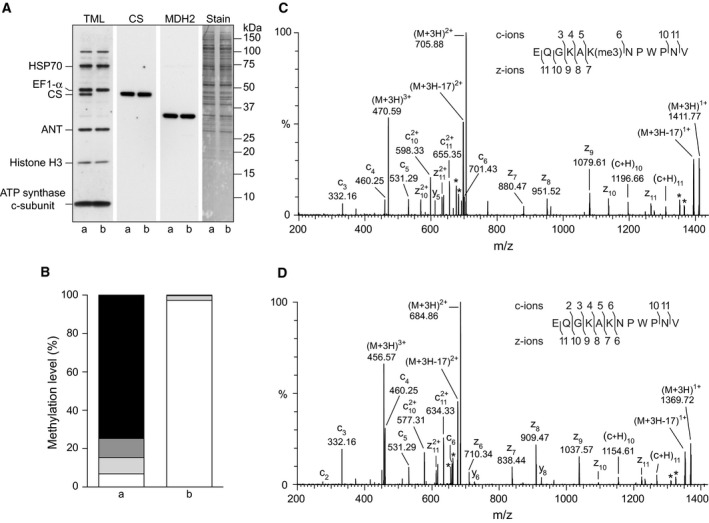
Effect of disruption of *METTL12* on the methylation of human citrate synthase. (A) Mitoplasts from a, wild‐type HAP1 cells and b, HAP1‐ΔMETTL12 cells, fractionated by SDS/PAGE and western blotted with antibodies against trimethyllysine (TML) and citrate synthase (CS). An antibody against malate dehydrogenase (MDH2), which is found in the matrix of mitochondria, provided a loading control. On the right‐hand side, the same membrane stained with Coomassie Blue dye. (B) The relative peak areas obtained from the extracted ion chromatograms of the various methylation states of the AspN peptide representing residues 363–374 of citrate synthase. The peptide was produced by digestion of the appropriate band in the fractionated mitoplast samples. Columns a and b, respectively, wild‐type and HAP1‐ΔMETTL12 cells. For the meaning of the various shades, see the legend to Fig. 1. (C,D) ETD fragmentation spectra of the AspN peptide from residues 363–374 from citrate synthase immunocaptured from mitochondria from HAP1 wild‐type and HAP1‐ΔMETTL12 cells; they represent the trimethylated and unmethylated forms of the peptide respectively. The asterisks indicate ions arising from neutral losses of CH_3_ NO and C_2_H_5_ NO from Gln and Asn residues. In the insets, fragment ions have been mapped onto the amino acid sequence.

The 240 amino acids of the mitochondrial precursor of human METTL12 are encoded by a single human gene on chromosome 11 with three exons interrupted by two introns. In the near‐haploid human cell line HAP1‐ΔMETTL12, exon III has been disrupted by the insertion of a single base. This insertion introduces a frame‐shift, leading to a truncated version of METTL12 with the N‐terminal mitochondrial import sequence from residues 1–21 29 and residues 1–30 of the mature protein, plus unrelated protein sequence terminated 66 amino acids later. Analysis of the mitoplasts from wild‐type HAP1 cells, revealed the expected presence of the three prominent mitochondrial proteins containing trimethyllysine residues, namely citrate synthase, the ADP/ATP translocase and the c‐subunit of ATP synthase. Similarly, the mitoplasts from HAP1‐ΔMETTL12 cells also contained the trimethylated ADP/ATP translocase and the c‐subunit of ATP synthase, and the same level of citrate synthase as wild‐type cells, but in contrast to the wild‐type cells, the HAP1‐ΔMETTL12 cells were devoid of the trimethylated form of citrate synthase (Fig. 2A). Both samples also contained additional bands, corresponding to contaminant nonmitochondrial HSP70 proteins and elongation factor 1α, which also contain trimethylated lysines 46, 47.

The methylation of Lys‐368 of citrate synthase in wild‐type HAP1 cells was verified by mass spectrometric analysis of the AspN peptide from residues 363–374. This analysis revealed that 74% of the peptide was trimethylated, 10% was dimethylated, 9% was monomethylated and 7% was unmethylated (Fig. 2 and Table S6). In contrast, in HAP1‐ΔMETTL12 cells, the level of the unmethylated peptide was 97% (Fig. 2 and Table S6). The residual 3% was predominantly monomethylated, probably arising from artefactual methyl esterification of residue Glu‐363 by acidic methanol used in the detection of the protein in the SDS/PAGE gel 48. Spectra arising from the fragmentation of triply charged ions of this peptide with *m*/*z* values of 456.57 and 470.59, confirmed the presence of unmethylated forms of Lys‐368 in HAP1‐ΔMETTL12 cells and of trimethylated forms in HAP1 parental cells (Fig. 2).

### Impact of the absence of METTL12 on HAP1 cells

**Figure 3:**
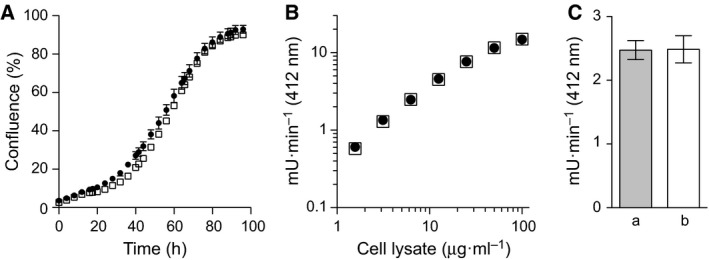
Comparison of properties of wild‐type HAP1 cells and HAP1‐ΔMETTL12 cells. (A) Growth rates. Initially, ca. 10^5^ HAP1 wild‐type cells (●) and 10^5^ HAP1‐ΔMETTL12 cells (□) were seeded into six‐well plates, and their confluence was measured over 96 h. The data points represent the mean values ± SD (*n* = 3). (B) Activity of citrate synthase in cell lysates from wild‐type cells (●) and HAP1‐ΔMETTL12 cells (□) (*n* = 3, SD values are smaller than the symbols). (C) Activity of citrate synthase in 6.25 μg·mL^−1^ of cell lysates from wild‐type cells (a) and HAP1‐ΔMETTL12 cells (b) (*n* = 3, ±SD). The concentration of citrate synthase in wild‐type and mutant cells did not differ significantly.

Under the limited range of conditions that were investigated, the removal of METTL12 had no impact on the proliferation of HAP1 cells (Fig. 3A). Additionally, neither the amount (wild‐type, 0.13 ± 0.03 and HAP1‐ΔMETTL12 0.14 ± 0.02 ng·μg^−1^ of cell lysate), nor the enzymic activity of citrate synthase (Fig. 3) was affected.

## Discussion

### Subcellular site of methylation of mitochondrial proteins

**Figure 4:**
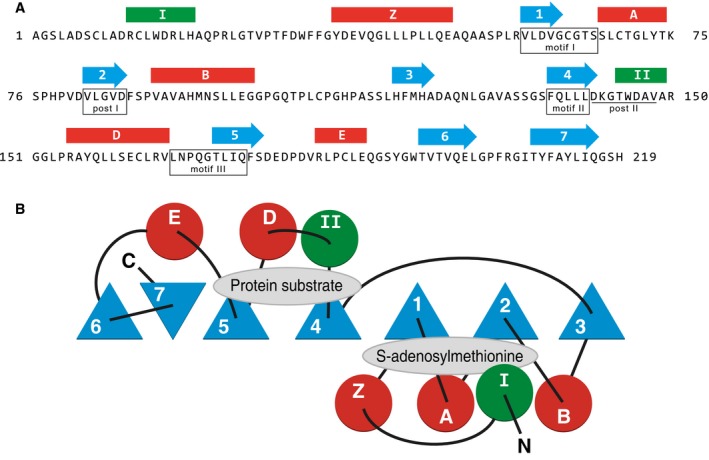
The predicted structure of human METTL12. (A) Predicted secondary structure of mature METTL12. The locations of α‐helices indicated above the sequence by red or green bars, and β‐sheets by blue arrows. Helix Z, β‐sheets 1‐7 and intervening α‐helices A‐E characterize the 7β‐strand methyltransferase fold. Helix C, intervening between strands 3 and 4 in the canonical fold for this family, is not present in METTL12. The additional predicted helices I and II are found in the structure of the related methyltransferase domain of endothelin‐converting enzyme‐2 65. Helix I is positioned over the fold. Conserved motifs I, post‐I, II and III are also found in 7β‐strand methyltransferases 66. The post‐II motif sequence (underlined) is highly conserved in other related methyltransferases METTL10, METTL13 and the methyltransferase domain of endothelin‐converting enzyme‐2. (B) Topology of the 7β‐strand fold and flanking α‐helices. Triangles, circles and lines represent β‐strands, α‐helices and joining loops respectively. The positions of the substrate binding sites are shown.

Nuclear‐encoded mitochondrial proteins are synthesized in the cytoplasm and are transferred to the organelle via TOM and TIM complexes in the outer and inner membranes respectively 38. Thus, it is conceivable that the post‐translational introduction of methyl groups into specific mitochondrial proteins by methyltransferases could occur at any stage during their transit to their final destinations in the organelle. Previously, in human mitochondria, three methyltransferases (HEMK1, NDUFAF7, METTL20), with SAM as the methyl donor, have been found uniquely in the matrix space (Table 1) 11, 21, 23. In addition, NDUFAF5, which probably uses SAM as a cofactor in the introduction of a hydroxyl group into the side chain of Arg‐73 in the NDUFS7 subunit of complex I, is found in the same subcellular location 24. METTL12, also located in the mitochondrial matrix, adds a fourth protein‐modifying methyltransferase to this growing list. Its predicted secondary structure indicates that it is a 7β‐strand methyltransferase with five of the six associated canonical α‐helices (Fig. 4). METTL12 has been placed into a subfamily of 7β‐strand methyltransferases, with human METTL10, METTL13 and ECE2, and yeast EFM4 25. They share regions of sequence homology including a postmotif‐II sequence that interacts with the specific substrate 49. Like METTL12, both METTL10 and EFM4 are lysine methyltransferases 50, 51. Therefore, it is likely that members of this subfamily all catalyse the methylation of lysine residues.

### The site of methylation in citrate synthase

**Figure 5:**
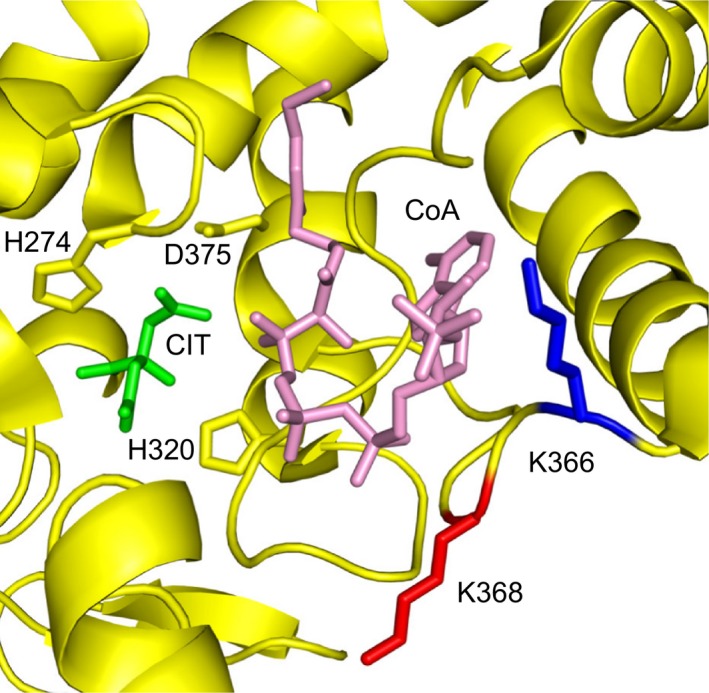
Location of methylated lysine‐368 in citrate synthase. The image, made with PyMol and shown in cartoon representation, displays the active site of one monomer of the dimeric porcine enzyme (PDB 2CTS). The CoA molecule is pink, and the side chain of the trimethylated Lys‐368 is red. The side chain of unmethylated Lys‐366 is blue. In addition to CoA, the active site also contains citrate (CIT, green). Amino acids H274, H320 and D375 participate in catalysis.

The three‐dimensional structure of human citrate synthase has not been determined, but the sequences of the human and porcine enzymes are 98% identical, and therefore the structure of the porcine enzyme 52 provides an excellent model for the human orthologue. It is known that, as in the human enzyme, Lys‐368 in the porcine protein is trimethylated 10. The active enzyme is a homodimer, and Lys‐368 is exposed on the surface of the enzyme in a loop in an extended structure that forms part of the binding pocket for CoA (Fig. 5). Lys‐366, nearby, is unmodified. Despite the modified residue being close to the active site of the enzyme, removal of the methyl group had no effect on the activity of the human enzyme *in vitro* (Fig. 3), and recombinant porcine citrate synthase lacking the methylation had a similar specific activity to the methylated enzyme isolated from a natural source 53. However, this is unlikely to reflect the *in vivo* situation in the mitochondrial matrix, where citrate synthase does not function in isolation, but as a component of the TCA cycle of enzymes. Moreover, while the removal of the methyl groups in HAP1‐ΔMETTL12 cells had no effect on cellular growth under the conditions that were investigated (Fig. 3); in the high‐glucose IMDM media, HAP1 cells can use aerobic glycolysis to generate the ATP and metabolites needed for growth. Therefore, under these conditions, the impact of any altered activity of citrate synthase on cell growth will be minimal.

**Figure 6:**
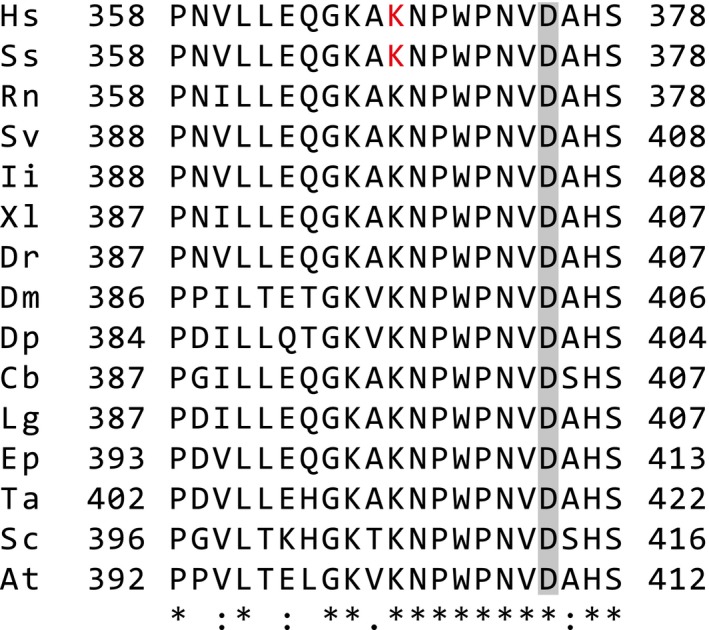
Conservation of lysine‐368 in citrate synthase. Alignment of sequences adjacent to human Lys‐368 from representative species across metazoans, in yeast and in a plant. The trimethyl‐lysines in the human and porcine proteins are red, and the grey box highlights a conserved residue in the catalytic triad of the enzyme. Hs, *Homo sapiens*; Ss, *Sus scrofa*; Rn, *Rattus norvegicus*; Sv, *Sturnus vulgaris*; Ii, *Iguana iguana*; Xl, *Xenopus laevis*; Dr, *Danio rerio*; Dm, *Drosophila melanogaster*; Dp, *Daphnia pulex*; Cb, *Caenorhabditis briggsae*; Lg, *Lottia gigantean*; Ep, *Exaiptasia pallida*; Ta, *Trichoplax adhaerens*; Sc, *Saccharomyces cerevisiae*; At, *Arabidopsis thaliana*. The alignment was made with Clustal Omega. The conserved human, porcine and rat proteins are numbered according to the mature porcine protein, after removal on the N‐terminal 27 amino acid mitochondrial targeting peptide 10. The numbering of all other proteins is based on the full‐length sequence. The symbols *, **:** and **.** denote identical, strongly conserved and weakly conserved residues respectively.

In contrast to the observed methylation, there was no evidence for the acetylation of lysine residues of citrate synthase in the mass spectrometric analysis of AspN or tryptic peptides (Tables S1–S4 and S7–S10). The equivalent concentrations of citrate synthase measured in HAP1 cells, despite there being significantly different levels of methylation, suggest that the modification does not influence the stability of the enzyme. However, the central location of the methylated site, its conservation throughout metazoans, and also in fungi and plants (Fig. 6), and the conservation of METTL12 across metazoans suggests that the modification is likely to be biologically significant.

Citrate synthase catalyses the first step of the tricarboxylic acid cycle, and there is extensive evidence that it forms a metabolon or metabolons with other citric acid cycle enzymes to facilitate the channelling of substrates between enzymes in the same pathway 54, 55, 56, 57. One proposal is that all of the enzymes that perform the cycle, plus aspartate aminotransferase and nucleoside diphosphate kinase, are organized around the α‐ketoglutarate dehydrogenase complex 58. Therefore, one possibility is that under particular cellular conditions, as yet undefined, the methylation of Lys‐368 regulates contacts with other enzymes in a metabolon of part or all of the citric acid cycle.

### Roles of methylation of mitochondrial proteins

Methylated proteins in mitochondria fall into two broad categories. In the first are ETFβ, where lysine residues 199 and 202 are methylated 11, 45, and citrate synthase described here. A characteristic of these proteins is that the extent of methylation is partial, and in the case of at least ETFβ, it varies according to growth conditions. In ETFβ, the modified site is adjacent to the recognition loop of the ETF, which has the remarkable property of recognizing and binding to as many as 13 different acyl‐CoA dehydrogenases so as to allow electron transfer between the dehydrogenases and the ETF 59, 60. It has been postulated that methylation of ETFβ helps to regulate these protein–protein interactions 11, 45, similar to one of the proposals being made here to explain the methylation of citrate synthase. Unlike regulation of pathways in other subcellular locations of human cells, there is no evidence in mitochondria that methylation is a reversible process, and so far, no mitochondrial protein demethylase has been identified. The second category consists of proteins where the site of methylation is modified completely, as in Arg‐85 of the NDUFS2 subunit of complex I 14, ADP/ATP translocase 12 and the c‐subunit of the ATP synthase complex 13. To this category, the complex I subunit, NDUFS7, can be added, where Arg‐73 is completely hydroxylated by NDUFAF5 14, 24. In the cases of NDUFS2 and NDUFS7, the modifications are absolutely required in the process of assembly of complex I, and, in each case, the absence of the modification stalls the assembly process at specific intermediate stages 23, 24, 61, 62. Therefore, it is possible that, in the ADP/ATP translocase and the c‐subunit of the ATP synthase, the complete methylation of the modified residues is also an obligatory step in the biogenesis and/or assembly of the proteins. The identification of the modifying enzymes would help in the testing of this hypothesis.

## Author contributions

JEW designed research and supervised project and prepared the manuscript; VFR, JC, SD and IMF performed research; VFR, JC, SD, IMF and JEW analysed data.

## Supporting information


**Table S1.** Protein identification of citrate synthase from HEK293T cells overexpressing METTL12.
**Table S2.** Protein identification of citrate synthase from HEK293T cells overexpressing METTL20.
**Table S3.** Protein identification of citrate synthase from HEK293T cells overexpressing METTL12.
**Table S4.** Protein identification of citrate synthase from HEK293T cells overexpressing METTL20.
**Table S5.** Quantitation of lysine 368 methylation levels of citrate synthase in HEK293T cells.
**Table S6.** Quantitation of lysine 368 methylation levels of citrate synthase in HAP1 cells.
**Table S7.** Protein identification of citrate synthase from HAP1 wild‐type cells.
**Table S8.** Protein identification of citrate synthase from HAP1 wild‐type cells.
**Table S9.** Protein identification of citrate synthase from HAP1‐ΔMETTL12 cells.
**Table S10.** Protein identification of citrate synthase from HAP1‐ΔMETTL12 cells.
