# Supplementary material for: Human METTL12 is a mitochondrial methyltransferase that modifies citrate synthase
Source: FEBS Lett. 2017 Apr 27;591(12):1641–52. doi: 10.1002/1873-3468.12649 (PMC5518231; doi:10.1002/1873-3468.12649)
Supplement: Supplementary file 1 — Table S1. Protein identification of citrate synthase from HEK293T cells overexpressing METTL12. Table S2. Protein identification of citrate synthase from HEK293T cells overexpressing METTL20. Table S3. Protein identification of citrate synthase from HEK293T cells overexpressing METTL12. Table S4. Protein identification of citrate synthase from HEK293T cells overexpressing METTL20. Table S5. Quantitation of lysine 368 methylation levels of citrate synthase in HEK293T cells. Table S6. Quantitation of lysine 368 methylation levels of citrate synthase in HAP1 cells. Table S7. Protein identification of citrate synthase from HAP1 wild‐type cells. Table S8. Protein identification of citrate synthase from HAP1 wild‐type cells. Table S9. Protein identification of citrate synthase from HAP1‐ΔMETTL12 cells. Table S10. Protein identification of citrate synthase from HAP1‐ΔMETTL12 cells. [file FEB2-591-1641-s001.pdf]

Supporting information for:

Human METTL12 is a mitochondrial methyltransferase that modifies citrate synthase

Virginie F. Rhein[1], Joe Carroll[1], Shujing Ding, Ian M. Fearnley and John E. Walker

Medical Research Council Mitochondrial Biology Unit, University of Cambridge, Cambridge Biomedical Campus, Cambridge, UK

[1] These authors contributed equally

Table S1. Protein identification of citrate synthase from HEK293T cells overexpressing METTL12.

The data were obtained by tandem mass spectrometric analysis of AspN derived peptides, using CID, in a LTQ OrbiTrap XL-electron transfer dissociation mass spectrometer. The peptide score is the Mascot ions score derived from a comparison with a database of human sequences (20,274 sequences, Uniprot 2015_07) with the following parameters: Peptide Mass Tolerance: ± 5 ppm; Fragment Mass Tolerance: ± 0.5 Da; Enzyme: Asp-N_ambic; Max Missed Cleavages: 3; Variable modifications: Oxidation (M), Propionamide (C), Trimethyl (K), Dimethyl (KR), Methyl (KR), Acetyl (K). Individual ion scores >35 indicate identity or extensive homology ($p<0.05$).

m/z	M observed	M calculated	ppm error	Score	Peptide sequence[a]
412.2228	822.4311	822.4310	0.21	9	T.EGLMKFV.D
488.7583	975.5021	975.5025	-0.44	38	E.EQVSWLSK.E
534.7952	1067.5759	1067.5764	-0.42	37	R.EFALKHLPN.D
449.2540	1344.7402	1344.7401	0.03	26	T.ELTRLYLTIHS.D
678.8981	1355.7816	1355.7813	0.23	68	Q.EVLVWLTQLQK.E
705.3803	1408.7459	1408.7463	-0.23	18	L.EQGKAKNPWPNV.D + Trimethyl (K)
777.8666	1553.7186	1553.7184	0.11	40	V.DAHSGVLLQYYGMT.E
793.3409	1584.6672	1584.6667	0.30	42	L.DWSHNFTNMLGYT.D
862.8855	1723.7564	1723.7554	0.60	35	V.DMMYGGMRGMKGLVY.E + Mox
870.4449	1738.8753	1738.8746	0.44	37	L.ERPKSMSTEGLMKFV.D
885.5123	1769.0101	1769.0087	0.79	46	Q.EVLVWLTQLQKEVGK.D
904.9959	1807.9773	1807.9767	0.32	31	K.EWAKRAALPSHVVTML.D
987.5084	1973.0022	1973.0007	0.76	19	T.DHQFTELTRLYLTIHS.D
987.9673	1973.9200	1973.9191	0.44	56	S.DHEGGNVSAHTSHLVGSALS.D
716.6924	2147.0555	2147.0548	0.33	41	S.ESNFARAYAQGISRTKYW.E
1074.5732	2147.1319	2147.1303	0.76	67	E.EPLPEGLFWLLVTGHIPTE.E
1109.1401	2216.2657	2216.2643	0.66	43	N.DPMFKLVAQLYKIVPNVLL.E + Mox
1164.5690	2327.1234	2327.1216	0.78	32	L.DNFPTNLHPMSQLSAAVTALNS.E
1164.5765	2327.1385	2327.1368	0.73	63	S.DPYLSFAAAMNGLAGPLHGLANQ.E + Mox
906.1495	2715.4268	2715.4245	0.84	43	R.DYIWNTLNSGRVVPGYGHAVLRKT.D

[a] Where both unmodified and modified (Mox) peptides were identified, and/or different peptide charge states, only the highest scoring peptide is included in the table. All peptides displayed are the highest ranked matches in the database (Rank 1 in the MASCOT output). Propionamide arises from reaction of cysteine residues with acrylamide, Mox is oxidized methionine. Full stops in the amino acid sequence denote the sites of cleavage.

Table S2. Protein identification of citrate synthase from HEK293T cells overexpressing METTL20.

The data were obtained by tandem mass spectrometric analysis of AspN derived peptides, using CID, in a LTQ OrbiTrap XL-electron transfer dissociation mass spectrometer. The peptide score is the Mascot ions score derived from a comparison with a database of human sequences (20,274 sequences, Uniprot 2015_07) with the following parameters: Peptide Mass Tolerance: ± 5 ppm; Fragment Mass Tolerance: ± 0.5 Da; Enzyme: Asp-N_ambic; Max Missed Cleavages: 3; Variable modifications: Oxidation (M), Propionamide (C), Trimethyl (K), Dimethyl (KR), Methyl (KR), Acetyl (K). Individual ion scores >35 indicate identity or extensive homology ($p<0.05$).

m/z	M observed	M calculated	ppm error	Score	Peptide sequence[a]
412.2229	822.4312	822.4310	0.28	23	T.EGLMKFV.D
488.7584	975.5022	975.5025	-0.37	39	E.EQVSWLSK.E
534.7952	1067.5759	1067.5764	-0.42	39	R.EFALKHLPN.D
449.2539	1344.7400	1344.7401	-0.10	27	T.ELTRLYLTIHS.D
678.8981	1355.7816	1355.7813	0.23	72	Q.EVLVWLTQLQK.E
684.3571	1366.6997	1366.6993	0.26	38	L.EQGKAKNPWPNV.D
691.3650	1380.7155	1380.7150	0.41	17	L.EQGKAKNPWPNV.D + Methyl (KR)
705.3807	1408.7469	1408.7463	0.46	23	L.EQGKAKNPWPNV.D + Trimethyl (K)
777.8670	1553.7194	1553.7184	0.66	33	V.DAHSGVLLQYYGMT.E
793.3410	1584.6674	1584.6667	0.45	24	L.DWSHNFTNMLGYT.D
862.8854	1723.7563	1723.7554	0.53	29	V.DMMYGGMRGMKGLVY.E + Mox
870.4449	1738.8752	1738.8746	0.36	39	L.ERPKSMSTEGLMKFV.D
885.5120	1769.0095	1769.0087	0.44	63	Q.EVLVWLTQLQKEVGK.D
904.9961	1807.9776	1807.9767	0.52	27	K.EWAKRAALPSHVVTML.D
987.5085	1973.0024	1973.0007	0.88	32	T.DHQFTELTRLYLTIHS.D
987.9675	1973.9204	1973.9191	0.63	59	S.DHEGGNVSAHTSHLVGSALS.D
716.6924	2147.0553	2147.0548	0.24	41	S.ESNFARAYAQGISRTKYW.E
1074.5735	2147.1324	2147.1303	0.99	51	E.EPLPEGLFWLLVTGHIPTE.E
1101.1428	2200.2711	2200.2693	0.79	54	N.DPMFKLVAQLYKIVPNVLL.E
1139.0946	2276.1746	2276.1729	0.78	53	G.EEPLPEGLFWLLVTGHIPTE.E
1164.5689	2327.1231	2327.1216	0.67	55	L.DNFPTNLHPMSQLSAAVTALNS.E
1172.5742	2343.1339	2343.1317	0.92	54	S.DPYLSFAAAMNGLAGPLHGLANQ.E + Mox
842.1423	2523.4050	2523.4034	0.63	17	K.EQARIKTFRQQHGKTVVGQITV.D
906.1497	2715.4272	2715.4245	0.97	46	R.DYIWNTLNSGRVVPGYGHAVLRKT.D
680.1196	2716.4492	2716.4483	0.34	14	P.DEGIRFRGFSIPECQKLLPKAKGG.E + Propionamide (C)

[a] Where both unmodified and modified (Mox) peptides were identified, and/or different peptide charge states, only the highest scoring peptide is included in the table. All peptides displayed are the highest ranked matches in the database (Rank 1 in the MASCOT output). Propionamide arises from reaction of cysteine residues with acrylamide, Mox is oxidized methionine. Full stops in the amino acid sequence denote the sites of cleavage.

Table S3. Protein identification of citrate synthase from HEK293T cells overexpressing METTL12.

The data were obtained by tandem mass spectrometric analysis of trypsin derived peptides, using CID, in a LTQ OrbiTrap XL-electron transfer dissociation mass spectrometer. The peptide score is the Mascot ions score derived from a comparison with a database of human sequences (20,274 sequences, Uniprot 2015_07) with the following parameters: Peptide Mass Tolerance: ± 5 ppm; Fragment Mass Tolerance: ± 0.5 Da; Enzyme: Trypsin; Max Missed Cleavages: 2; Variable modifications: Oxidation (M), Propionamide (C), Trimethyl (K), Dimethyl (KR), Methyl (KR), Acetyl (K). Individual ion scores >32 indicate identity or extensive homology ($p<0.05$).

m/z	M observed	M calculated	ppm error	Score	Peptide sequence[a]
417.7580	833.5014	833.5011	0.36	31	K.LVAQLYK.I
492.2303	982.4461	982.4463	-0.19	30	K.SMSTEGLMK.F
499.3002	996.5858	996.5855	0.26	42	K.DILADLIPK.E
549.7738	1097.5330	1097.5328	0.23	47	K.HLPNDPMFK.L
564.3323	1126.6500	1126.6499	0.13	42	R.ALGFPLERPK.S
584.3356	1166.6566	1166.6560	0.47	26	R.VVPGYGHAVLR.K
605.3640	1208.7135	1208.7129	0.50	42	K.IVPNVLLEQGK.A
663.8987	1325.7828	1325.7819	0.66	84	R.ALGVLAQLIWSR.A
669.8259	1337.6372	1337.6364	0.59	52	R.DYIWNTLNSGR.V
881.9472	1761.8798	1761.8785	0.77	49	K.GLVYETSVLDPDEGIR.F
887.4213	1772.8280	1772.8260	1.12	85	K.TVVGQITVDMMYGGMR.G + Mox
1276.2520	3825.7340	3825.7329	0.29	56	R.EGSGIGAIDSNLDWSHNFTNMLGY TDHQFTELTR.L
1384.6947	4151.0623	4151.0568	1.32	46	R.AALPSHVVTMLDNFPTNLHPMSQL SAAVTALNSESNFAR.A

[a] Where both unmodified and modified (Mox) peptides were identified, and/or different peptide charge states, only the highest scoring peptide is included in the table. All peptides displayed are the highest ranked matches in the database (Rank 1 in the MASCOT output). Propionamide arises from reaction of cysteine residues with acrylamide, Mox is oxidized methionine. Full stops in the amino acid sequence denote the sites of cleavage.

Table S4. Protein identification of citrate synthase from HEK293T cells overexpressing METTL20.

The data were obtained by tandem mass spectrometric analysis of trypsin derived peptides, using CID, in a LTQ OrbiTrap XL-electron transfer dissociation mass spectrometer. The peptide score is the Mascot ions score derived from a comparison with a database of human sequences (20,274 sequences, Uniprot 2015_07) with the following parameters: Peptide Mass Tolerance: ± 5 ppm; Fragment Mass Tolerance: ± 0.5 Da; Enzyme: Trypsin; Max Missed Cleavages: 2; Variable modifications: Oxidation (M), Propionamide (C), Trimethyl (K), Dimethyl (KR), Methyl (KR), Acetyl (K). Individual ion scores >31 indicate identity or extensive homology (p<0.05).

m/z	M observed	M calculated	ppm error	Score	Peptide sequence[a]
417.7580	833.5014	833.5011	0.36	31	K.LVAQLYK.I
433.2300	864.4454	864.4453	0.08	42	R.AYAQGISR.T
499.3000	996.5855	996.5855	-0.04	30	K.DILADLIPK.E
500.2278	998.4410	998.4413	-0.25	38	K.SMSTEGLMK.F + Mox
540.2631	1078.5116	1078.5117	-0.14	29	R.GFSIPECQK.L + Propionamide (C)
549.7737	1097.5329	1097.5328	0.12	36	K.HLPNDPMFK.L
564.3323	1126.6500	1126.6499	0.13	38	R.ALGFPLERPK.S
584.3357	1166.6569	1166.6560	0.78	62	R.VVPGYGHAVLR.K
663.8988	1325.7830	1325.7819	0.84	57	R.ALGVLAQLIWSR.A
669.8259	1337.6372	1337.6364	0.59	54	R.DYIWNTLNSGR.V
879.4235	1756.8324	1756.8311	0.75	78	K.TVVGQITVDMMYGGMR.G
1276.2531	3825.7373	3825.7329	1.15	8	R.EGSGIGAIDSNLDWSHNFTNMLGY TDHQFTELTR.L
1046.7710	4183.0549	4183.0466	1.97	11	R.AALPSHVVTMLDNFPTNLHPMSQL SAAVTALNSESNFAR.A + 2 Mox

[a] Where both unmodified and modified (Mox) peptides were identified, and/or different peptide charge states, only the highest scoring peptide is included in the table. All peptides displayed are the highest ranked matches in the database (Rank 1 in the MASCOT output). Propionamide arises from reaction of cysteine residues with acrylamide, Mox is oxidized methionine. Full stops in the amino acid sequence denote the sites of cleavage.

Table S5. Quantitation of lysine 368 methylation levels of citrate synthase in HEK293T cells.

Cell	Peptide[1]	Peptide peak area[2]				Average % (sd)
		A	%	B	%	
Control	Me0	21380306	44.1	25891038	44.5	44.3 (0.3)
	Me1	12633898	26.1	15513065	26.7	26.4 (0.4)
	Me2	7293601	15.0	8234599	14.2	14.6 (0.6)
	Me3	7180056	14.8	8507996	14.6	14.7 (0.2)
METTL12	Me0	104668	0.2	73658	0.3	0.3 (0.0)
	Me1	44077	0.1	376120	1.5	0.8 (1.0)
	Me2	4186	0.0	7303	0.0	0.0 (0.0)
	Me3	41872811	99.6	25146475	98.2	98.9 (1.0)
METTL20	Me0	23088651	30.2	13451915	30.2	30.2 (0.0)
	Me1	17493216	22.9	11286569	25.4	24.1 (1.8)
	Me2	12780321	16.7	8304585	18.7	17.7 (1.4)
	Me3	23052597	30.2	11437303	25.7	27.9 (3.1)

[1] The data corresponds to the analysis of citrate synthase peptide (363)EQGKAKNPWPNV(374) and the methylated derivatives of Lys-368. Me0, unmodified; Me1, monomethyl; Me2, dimethyl; Me3, trimethyl. Residue numbers refer to the mature human citrate synthase protein sequence. The peptide was obtained from an in-gel AspN digest of gel sections containing citrate synthase, excised from an SDS-PAGE separation of mitoplast proteins.

[2] The data was obtained from two samples (A and B). Peak areas were derived from extracted ion chromatograms, using Gaussian smoothing and a m/z tolerance of 5 ppm. Monoisotopic m/z values used were 684.3569 (M+2H) and 456.5737 (M+3H) for the non-methylated peptide, with 14.0156 Da added for each methyl group respectively. Data from both 2+ and 3+ ions of each peptide were summed. Relative peak areas were calculated for each analysis, followed by the average and standard deviation (sd) of the samples.

Table S6. Quantitation of lysine 368 methylation levels of citrate synthase in HAP1 cells.

Cell	Peptide[1]	Peptide peak area[2]				Average % (sd)
		A	%	B	%	
Wild-type	Me0	3507339	6.3	2899266	7.5	6.9 (0.8)
	Me1	4664664	8.4	3671762	9.5	9.0 (0.8)
	Me2	5157855	9.3	4053881	10.5	9.9 (0.9)
	Me3	41994438	75.9	27824007	72.4	74.1 (2.5)
ΔMETTL12	Me0	47946009	95.4	110480663	98.1	96.8 (1.9)
	Me1	2294022	4.6	1292026	1.1	2.9 (2.4)
	Me2	4637	0.0	98103	0.1	0.0 (0.1)
	Me3	2420	0.0	716755	0.6	0.3 (0.4)

[1] The data corresponds to the analysis of citrate synthase peptide (363)EQGKAKNPWPNV(374) and the methylated derivatives of Lys-368. Me0, unmodified; Me1, monomethyl; Me2, dimethyl; Me3, trimethyl. Residue numbers refer to the mature human citrate synthase protein sequence. The peptide was obtained from an in-gel AspN digest of gel sections containing citrate synthase, excised from an SDS-PAGE separation of mitoplast proteins.

[2] The data was obtained from two samples (A and B). Peak areas were derived from extracted ion chromatograms, using Gaussian smoothing and a m/z tolerance of 5 ppm. Monoisotopic m/z values used were 684.3569 (M+2H) and 456.5737 (M+3H) for the non-methylated peptide, with 14.0156 Da added for each methyl group respectively. Data from both 2+ and 3+ ions of each peptide were summed. Relative peak areas were calculated for each analysis, followed by the average and standard deviation (sd) of the samples.

Table S7. Protein identification of citrate synthase from HAP1 wild-type cells.

The data were obtained by tandem mass spectrometric analysis of AspN derived peptides, using CID, in a LTQ OrbiTrap XL-electron transfer dissociation mass spectrometer. The peptide score is the Mascot ions score derived from a comparison with a database of human sequences (20,274 sequences, Uniprot 2015_07) with the following parameters: Peptide Mass Tolerance: ± 5 ppm; Fragment Mass Tolerance: ± 0.5 Da; Enzyme: Asp-N_ambic; Max Missed Cleavages: 3; Variable modifications: Oxidation (M), Propionamide (C), Trimethyl (K), Dimethyl (KR), Methyl (KR), Acetyl (K). Individual ion scores >34 indicate identity or extensive homology (p<0.05).

m/z	M observed	M calculated	ppm error	Score	Peptide sequence[a]
488.7580	975.5014	975.5025	-1.19	40	E.EQVSWLSK.E
534.7950	1067.5754	1067.5764	-0.89	35	R.EFALKHLPN.D
673.3765	1344.7385	1344.7401	-1.20	40	T.ELTRLYLTIHS.D
691.3633	1380.7120	1380.7150	-2.15	16	L.EQGKAKNPWPNV.D + Methyl (KR)
698.3710	1394.7274	1394.7306	-2.32	21	L.EQGKAKNPWPNV.D + Dimethyl (KR)
705.3791	1408.7436	1408.7463	-1.88	28	L.EQGKAKNPWPNV.D + Trimethyl (K)
785.8622	1569.7098	1569.7133	-2.26	44	V.DAHSGVLLQYYGMT.E + Mox
801.3363	1600.6580	1600.6617	-2.25	24	L.DWSHNFTNMLGYT.D + Mox
854.8859	1707.7572	1707.7605	-1.95	25	V.DMMYGGMRGMKGLVY.E
870.4424	1738.8703	1738.8746	-2.45	40	L.ERPKSMSTEGLMKFV.D
885.5096	1769.0046	1769.0087	-2.32	53	Q.EVLVWLTQLQKEVGK.D
987.5046	1972.9947	1973.0007	-3.01	44	T.DHQFTELTRLYLTIHS.D
987.9650	1973.9154	1973.9191	-1.9	56	S.DHEGGNVSAHTSHLVGSALS.D
716.6910	2147.0511	2147.0548	-1.71	19	S.ESNFARAYAQGISRTKYW.E
1074.5702	2147.1258	2147.1303	-2.08	52	E.EPLPEGLFWLLVTGHIPTE.E
1101.1398	2200.2650	2200.2693	-1.98	54	N.DPMFKLVAQLYKIVPNVLL.E
1139.0914	2276.1683	2276.1729	-2.01	54	G.EEPLPEGLFWLLVTGHIPTE.E
1164.5641	2327.1136	2327.1216	-3.42	62	L.DNFPTNLHPMSQLSAAVTALNS.E
1172.5697	2343.1248	2343.1317	-2.94	59	S.DPYLSFAAAMNGLAGPLHGLANQ.E + Mox
906.1462	2715.4169	2715.4245	-2.81	32	R.DYIWNTLNSGRVVPGYGHAVLRKT.D
992.5524	2974.6355	2974.6426	-2.39	12	M.DLIAKLPCVAAKIYRNLYREGSGIGAI.D + Propionamide (C)

[a] Where both unmodified and modified (Mox) peptides were identified, and/or different peptide charge states, only the highest scoring peptide is included in the table. All peptides displayed are the highest ranked matches in the database (Rank 1 in the MASCOT output). Propionamide arises from reaction of cysteine residues with acrylamide, Mox is oxidized methionine. Full stops in the amino acid sequence denote the sites of cleavage.

Table S8. Protein identification of citrate synthase from HAP1 wild-type cells.

The data were obtained by tandem mass spectrometric analysis of trypsin derived peptides, using CID, in a LTQ OrbiTrap XL-electron transfer dissociation mass spectrometer. The peptide score is the Mascot ions score derived from a comparison with a database of human sequences (20,274 sequences, Uniprot 2015_07) with the following parameters: Peptide Mass Tolerance: ± 5 ppm; Fragment Mass Tolerance: ± 0.5 Da; Enzyme: Trypsin; Max Missed Cleavages: 2; Variable modifications: Oxidation (M), Propionamide (C), Trimethyl (K), Dimethyl (KR), Methyl (KR), Acetyl (K). Individual ion scores >31 indicate identity or extensive homology ($p<0.05$).

m/z	M observed	M calculated	ppm error	Score	Peptide sequence[a]
417.7579	833.5013	833.5011	0.29	28	K.LVAQLYK.I
492.2303	982.4460	982.4463	-0.31	49	K.SMSTEGLMK.F
499.2999	996.5852	996.5855	-0.28	44	K.DILADLIPK.E
500.2274	998.4403	998.4413	-0.97	9	K.SMSTEGLMK.F + Mox
540.2630	1078.5114	1078.5117	-0.25	33	R.GFSIPECQK.L + Propionamide (C)
549.7731	1097.5316	1097.5328	-1.10	19	K.HLPNDPMFK.L
564.3315	1126.6485	1126.6499	-1.18	40	R.ALGFPLERPK.S
605.3632	1208.7119	1208.7129	-0.80	38	K.IVPNVLLEQGK.A
648.3818	1294.7491	1294.7510	-1.44	4	R.VVPGYGHAVLRK.T
663.8973	1325.7800	1325.7819	-1.45	69	R.ALGVLAQLIWSR.A
669.8249	1337.6353	1337.6364	-0.78	57	R.DYIWNTLNSGR.V
804.4166	1606.8187	1606.8216	-1.78	51	K.LRDYIWNTLNSGR.V
879.4208	1756.8271	1756.8311	-2.24	111	K.TVVGQITVDMMYGGMR.G
952.9503	1903.8860	1903.8913	-2.81	70	K.YWELIYEDSMDLIAK.L + Mox
638.6812	1913.0216	1913.0233	-0.88	17	K.HLPNDPMFKLVAQLYK.I
1059.5238	2117.0330	2117.0390	-2.83	81	R.TKYWELIYEDSMDLIAK.L
1116.9072	3347.6999	3347.7078	-2.37	33	K.GGEEPLPEGLFWLLVTGHIPTEEQV SWLSK.E
1276.2477	3825.7212	3825.7329	-3.06	56	R.EGSGIGAIDSNLDWSHNFTNMLGY TDHQFTELTR.L
1384.6882	4151.0429	4151.0568	-3.36	37	R.AALPSHVVTMLDNFPTNLHPMSQL SAAVTALNSESNFAR.A

[a] Where both unmodified and modified (Mox) peptides were identified, and/or different peptide charge states, only the highest scoring peptide is included in the table. All peptides displayed are the highest ranked matches in the database (Rank 1 in the MASCOT output). Propionamide arises from reaction of cysteine residues with acrylamide, Mox is oxidized methionine. Full stops in the amino acid sequence denote the sites of cleavage.

Table S9. Protein identification of citrate synthase from HAP1-ΔMETTL12 cells.

The data were obtained by tandem mass spectrometric analysis of AspN derived peptides, using CID, in a LTQ OrbiTrap XL-electron transfer dissociation mass spectrometer. The peptide score is the Mascot ions score derived from a comparison with a database of human sequences (20,274 sequences, Uniprot 2015_07) with the following parameters: Peptide Mass Tolerance: ± 5 ppm; Fragment Mass Tolerance: ± 0.5 Da; Enzyme: Asp-N_ambic; Max Missed Cleavages: 3; Variable modifications: Oxidation (M), Propionamide (C), Trimethyl (K), Dimethyl (KR), Methyl (KR), Acetyl (K). Individual ion scores >34 indicate identity or extensive homology (p<0.05).

m/z	M observed	M calculated	ppm error	Score	Peptide sequence[a]
412.2228	822.4311	822.4310	0.14	33	T.EGLMKFV.D
488.7585	975.5024	975.5025	-0.13	38	E.EQVSWLSK.E
534.7948	1067.5750	1067.5764	-1.22	34	R.EFALKHLPN.D
673.3763	1344.7380	1344.7401	-1.58	46	T.ELTRLYLTIHS.D
678.8974	1355.7802	1355.7813	-0.76	86	Q.EVLVWLTQLQK.E
684.3556	1366.6967	1366.6993	-1.89	34	L.EQGKAKNPWPNV.D
785.8626	1569.7107	1569.7133	-1.71	26	V.DAHSGVLLQYYGMT.E + Mox
801.3363	1600.6580	1600.6617	-2.25	33	L.DWSHNFTNMLGYT.D + Mox
861.9216	1721.8287	1721.8333	-2.66	27	H.EGGNVSAHTSHLVGSALS.D
862.8821	1723.7497	1723.7554	-3.3	32	V.DMMYGGMRGMKGLVY.E + Mox
870.4424	1738.8702	1738.8746	-2.52	41	L.ERPKSMSTEGLMKFV.D
885.5098	1769.0051	1769.0087	-2.04	55	Q.EVLVWLTQLQKEVGK.D
904.9937	1807.9727	1807.9767	-2.18	20	K.EWAKRAALPSHVVTML.D
987.5046	1972.9947	1973.0007	-3.01	24	T.DHQFTELTRLYLTIHS.D
987.9648	1973.9151	1973.9191	-2.03	52	S.DHEGGNVSAHTSHLVGSALS.D
716.6910	2147.0513	2147.0548	-1.63	37	S.ESNFARAYAQGISRTKYW.E
1074.5709	2147.1273	2147.1303	-1.4	47	E.EPLPEGLFWLLVTGHIPTE.E
1109.1368	2216.2591	2216.2643	-2.32	45	N.DPMFKLVAQLYKIVPNVLL.E + Mox
1139.0917	2276.1688	2276.1729	-1.8	61	G.EEPLPEGLFWLLVTGHIPTE.E
573.5815	2290.2968	2290.2983	-0.65	10	M.DLIAKLPCVAAKIYRNLYR.E + Propionamide (C)
1164.5654	2327.1163	2327.1216	-2.27	38	L.DNFPTNLHPMSQLSAAVTALNS.E
1172.5707	2343.1268	2343.1317	-2.11	72	S.DPYLSFAAAMNGLAGPLHGLANQ.E + Mox
906.1468	2715.4187	2715.4245	-2.13	40	R.DYIWNTLNSGRVVPGYGHAVLRKT.D
922.8277	2765.4613	2765.4687	-2.67	5	E.EQVSWLSKEWAKRAALPSHVVTML.D
712.3788	2845.4863	2845.4908	-1.61	17	P.DEGIRFRGFSIPECQKLLPKAKGGE.E + Propionamide (C)
1488.3232	2974.6319	2974.6426	-3.58	12	M.DLIAKLPCVAAKIYRNLYREGSGIGAI.D + Propionamide (C)
1227.9718	3680.8936	3680.9025	-2.42	30	S.DPYLSFAAAMNGLAGPLHGLANQEVLVWLTQLQK.E + Mox
1365.7129	4094.1168	4094.1299	-3.19	47	S.DPYLSFAAAMNGLAGPLHGLANQEVLVWLTQLQKEVGK.D + Mox

1119.0436	4472.1452	4472.1607	-3.47	18	L.DNFPTNLHPMSQLSAAVTALNSESN FARAYAQGISRTKYW.E + Mox

[a] Where both unmodified and modified (Mox) peptides were identified, and/or different peptide charge states, only the highest scoring peptide is included in the table. All peptides displayed are the highest ranked matches in the database (Rank 1 in the MASCOT output). Propionamide arises from reaction of cysteine residues with acrylamide, Mox is oxidized methionine. Full stops in the amino acid sequence denote the sites of cleavage.

Table S10. Protein identification of citrate synthase from HAP1-ΔMETTL12 cells.

The data were obtained by tandem mass spectrometric analysis of trypsin derived peptides, using CID, in a LTQ OrbiTrap XL-electron transfer dissociation mass spectrometer. The peptide score is the Mascot ions score derived from a comparison with a database of human sequences (20,274 sequences, Uniprot 2015_07) with the following parameters: Peptide Mass Tolerance: ± 5 ppm; Fragment Mass Tolerance: ± 0.5 Da; Enzyme: Trypsin; Max Missed Cleavages: 2; Variable modifications: Oxidation (M), Propionamide (C), Trimethyl (K), Dimethyl (KR), Methyl (KR), Acetyl (K). Individual ion scores >31 indicate identity or extensive homology ($p<0.05$).

m/z	M observed	M calculated	ppm error	Score	Peptide sequence[a]
417.7579	833.5013	833.5011	0.29	26	K.LVAQLYK.I
433.2299	864.4453	864.4453	0.01	43	R.AYAQGISR.T
492.2304	982.4463	982.4463	-0.01	46	K.SMSTEGLMK.F
499.3000	996.5854	996.5855	-0.10	55	K.DILADLIPK.E
500.2278	998.4411	998.4413	-0.13	56	K.SMSTEGLMK.F + Mox
540.2630	1078.5114	1078.5117	-0.25	33	R.GFSIPECQK.L + Propionamide (C)
549.7736	1097.5326	1097.5328	-0.2	34	K.HLPNDPMFK.L
564.3320	1126.6494	1126.6499	-0.42	37	R.ALGFPLERPK.S
605.3633	1208.7121	1208.7129	-0.61	41	K.IVPNVLLEQGK.A
663.8975	1325.7804	1325.7819	-1.18	70	R.ALGVLAQLIWSR.A
669.8247	1337.6347	1337.6364	-1.23	62	R.DYIWNTLNSGR.V
804.4152	1606.8159	1606.8216	-3.53	38	K.LRDYIWNTLNSGR.V
588.2998	1761.8776	1761.8785	-0.52	39	K.GLVYETSVLDPDEGIR.F
895.4150	1788.8154	1788.8209	-3.07	105	K.TVVGQITVDMMYGGMR.G + 2 Mox
952.9498	1903.8851	1903.8913	-3.27	85	K.YWELIYEDSMDLIAK.L + Mox
1059.5234	2117.0323	2117.039	-3.17	84	R.TKYWELIYEDSMDLIAK.L
1116.9076	3347.7009	3347.7078	-2.05	35	K.GGEEPLPEGLFWLLVTGHIPTEEQV SWLSK.E
1276.2476	3825.7209	3825.7329	-3.16	83	R.EGSGIGAIDSNLDWSHNFTNMLGY TDHQFTELTR.L
1390.0209	4167.0408	4167.0517	-2.63	61	R.AALPSHVVTMLDNFPTNLHPMSQL SAAVTALNSESNFAR.A + Mox

[a] Where both unmodified and modified (Mox) peptides were identified, and/or different peptide charge states, only the highest scoring peptide is included in the table. All peptides displayed are the highest ranked matches in the database (Rank 1 in the MASCOT output). Propionamide arises from reaction of cysteine residues with acrylamide, Mox is oxidized methionine. Full stops in the amino acid sequence denote the sites of cleavage.
